# The Streptokinase Therapy Complications and its Associated Risk Factors in Patients with Acute ST Elevation Myocardial Infarction

**Published:** 2018

**Authors:** Naser Aslanabadi, Naser Safaie, Fereshteh Talebi, Samaneh Dousti, Taher Entezari-Maleki

**Affiliations:** a *Cardiovascular Research Center, Tabriz University of Medical Sciences, Tabriz, Iran. *; b *Drug Applied Research Center, Tabriz University of Medical Sciences, Tabriz, Iran.*

**Keywords:** Streptokinase, Acute myocardial infarction (MI), Adverse drug reactions (ADRs), Hemorrhagic stroke, Allergy

## Abstract

Acute myocardial infarction (AMI) is one of the main leading causes of mortality and morbidity. Despite the progress in the treatment of AMI, streptokinase is still being used in many countries. Because of the critical condition of patients with AMI and complications of streptokinase therapy, this study was performed to evaluate the pattern of adverse drug reaction (ADRs) induced by streptokinase and its associated risk factors in patients with acute ST elevation MI.

A prospective cross-sectional study in a 14-month period was done at the university affiliated referral cardiovascular center. The Naranjo probability scale and Food and drug administration (FDA) criteria for severity of ADRs were performed for assessing the ADRs. The linear and logistic regression tests were used to evaluate the correlation between ADRs and study risk factors.

During the study period, 217 patients who received streptokinase were entered. The majority of patients (n = 191) experienced at least one ADR. Six patients died in-hospital mainly because of cardiac causes. The history of drug allergy was the main predictor in occurring of ADRs (Odds ratio: 3.26; 95% CI: 1.48-457.6; *p* =0.026). The most serious ADR was hemorrhagic stroke with a 1.4% incidence. Hypotension was one of the most occurred ADR (n = 75). Anaphylactic shock was not detected in this study.

In summary, our study showed that the history of drug allergy is the main predictor in occurring of ADRs by streptokinase. Furthermore, streptokinase therapy was associated with a higher rate of hemorrhagic stroke in Iranian population.

## Introduction

In 1998, adverse drug reactions (ADRs) were known as 4-6^th^ leading cause of mortality in the U.S with a cost for health care system (1). Furthermore, in the United Kingdom, data showed that 1 of 16 hospital admissions was associated to ADRs with the annually cost of up to £466 million. In addition, ADRs were responsible to 0.15% of in-hospital mortalities (2). Therefore, detection and prevention of ADRs play an important role in improvement of treatment outcomes.

Streptokinase, a metabolic product of beta-hemolytic streptococci, is an indirect fibrinolytic agent that interacts with plasminogen and forms an active complex with the protease activity that transforms plasminogen to plasmin (3). 

The mortality benefit of streptokinase in patients with acute myocardial infarction (AMI) has been illustrated in large, placebo-controlled trials (4, 5). 

In GISSI-1, the first largest thrombolytic trial evaluated streptokinase therapy versus no treatment in AMI patients, the significant mortality benefit of streptokinase has been documented as following: 8.2% versus 15.4% for patients received streptokinase within 1 hours of onset of symptoms, 9.2% versus 12% within three hours and 11.7% versus 14.1% in those treated between 3-6 h (4). In ISIS-2 trial, the similar benefits of streptokinase were also noted in over 17,000 patients presenting with symptom of acute MI within 24 h (5). Despite the mortality benefits of streptokinase in AMI patients, it includes some adverse reactions such as allergic reactions, hypotension, and bleeding. 

Streptokinase is a non-human protein, and its presentation into the circulatory system can lead to severe anaphylactic responses including death (6, 7). The risk of this immune response is dependent to the level of the anti-streptokinase antibodies in circulation. This immunogenicity limits multiple treatments with streptokinase (8, 9).

Because of the critical condition of patients with AMI and complications of streptokinase therapy, this study was performed to evaluate the pattern of adverse drug reactions induced by streptokinase therapy and its associated risk factors in patients with acute ST elevation MI (STEMI).

## Experimental

*Study design and setting*

A prospective cross-sectional study was performed in Shahid Madani Heart Center, the largest referral center for cardiovascular disorders at the north-west of Iran for a period of 14 months. The study was approved in the ethics committee of the university. 

*Sample size calculation*

Based on data from our center, about 400 patients with acute STEMI have been receiving thrombolytic therapy each year. Assuming the 95% confidence interval and α = 0.05 the sample size will be 210 patients with a standard error of 0.025 during 14-month study period.

*Inclusion and exclusion criteria*

The patients’ eligibility criteria included the age of over 18 years old with a diagnosis of acute STEMI who candidate for receiving streptokinase.

Exclusion criteria included acute or chronic kidney and liver disease, pregnant women and patients not able to continue the study, and contraindications of thrombolytic therapy including recent head/facial trauma and/or ischemic stroke within last 3 months, intracranial tumor, and prior intracranial hemorrhage, suspected aortic dissection, active internal bleeding, or bleeding diathesis and severe uncontrolled hypertension. 

*Streptokinase injection method*

The streptokinase was administered as soon as possible after the first symptoms of STEMI (under the six hours from onset of symptoms) with the usual adult dose of AMI as 1,500,000 units intravenous (IV) infusion over 60 min. In case of allergic reactions and fever, it was recommended that patients concurrently should receive corticosteroids that can be repeated during treatment. Before treatment, the patient PT and PTT were being controlled. In case of anaphylactic shock symptoms and hypotension, malaise, chills, nausea and arrhythmia, the infusion was being stopped.

*Detection and classification of ADRs*

All patients receiving streptokinase that had completed the informed consent form were monitored for ADRs induced by streptokinase. Detection and monitoring of ADRs were done through completing a questionnaire by reviewing the patientsꞌ medical file and documentation as well as interviewing with the patients.

The questionnaire includes the demographic information, past medical history, drug history, familial, habitual and social history, laboratory and echocardiographic information.

The Naranjo probability scale was used to causality assessment of ADRs as follows: The scores between 1-4 ranked as possible, 5-8 ranked as probable and > 9 ranked as highly probable. The score of < 0 was deemed as doubtful ADR (10). Based on the FDA classification, the severity of ADRs was categorized in four levels: 1) mild: no need to treatment, 2) moderate: need to specific treatment, 3) severe: cause to prolonged hospitalization, 4) very severe: potential life-threatening or contribute to the death.

*Data analysis*

Data analysis was performed using SPSS 16.0 (Chicago, SPSS Inc., 2007). The Kolmogorov-Smirnov test was utilized to determine the normally distribution of data. The correlation evaluation between ADRs and the study parameters was done using Spearman test. The linear regression (Stepwise method) and logistic regression analysis were performed to find out any relationships between the incidence and number of ADRs and the study independent risk factors. Continues data were presented as mean ± standard deviation (SD). *P*-values less than 0.05 were considered statistically significant.

## Results

During the 14-month study period, 217 patients who received streptokinase entered the study. The majority of patients were male (n = 178, 82%). The mean age of study population was 60.5 ± 12 years old. A total of 6 deaths occurred during the study period.

**Table 1.** The medical history of patients received streptokinase

**Characteristics**	**N (%)**
Ischemic heart disease	46 (21.2)
Cerebrovascular accident	6 (2.8)
Diabetes mellitus	58(26.9)
Ischemic heart disease	4 (20)
Hypertension	91 (42.1)
Hyperlipidemia Allergy Asthma Chronic obstructive pulmonary disease Kidney disorders Hepatitis B surface antigens +	52 (24.1) 4(1.8) 3 (1.4) 3 (1.4) 4 (1.8) 4 (1.8)
Other disease	25 (11.52)
Smoking	96 (44.2)
Alcohol	4 (1.8)
Addiction Bread making Coronary artery bypass graft Percutaneous coronary intervention	17 (7.8) 5 (2.3) 3 (1.4) 10 (4.6)
Myocardial infarction in families	19 (8.8)
Surgery	23 (10.59)
Cardiovascular drugs	77 (35.5)
Hyperlipidemia drugs	17 (7.8)
Non-steroidal anti-inflammatory drugs	4 (1.8)
Central nervous system drugs	11 (5.1)
Diabetic drugs	30 (12.4)

**Table 2:** The frequency of Streptokinase induced adverse drug reactions.

**Characteristics**	**N (%)**
Chest pain	82(37.9)
Hypotension	75(34.6)
Coughing	27(12.9)
Ecchymose	25(11.5)
Nausea and vomiting	22(10.2)
Pain in the extremities	22(10.2)
Bronchospasm	21(9.7)
Bradycardia	21(9.7)
Sweating	21(9.7)
Tachycardia	19(8.8)
Fever and chill	18(8.4)
Ventricular fibrillation	16(7.4)
Headache	13(6)
Hypertension	11(5.1)
Bleeding	11(5.1)
Agitatation	10(4.6)
Back pain	10(4.3)
Abdominal cramp	9(4.2)
Gastrointestinal bleeding	9(4.2)
Pulmonary edema	8(3.7)
Epigastric pain	8(3.7)
Constipation	8(3.7)
Hematuria	7(3.3)
Hemoptysis	7(3.3)
Allergy and erythema	6(2.8)
Phlebitis	6(2.8)
Cardiogenic shock	6(2.8)
Atrial fibrillation	5(2.3)
Swelling and edema in the extremities	4(1.9)
Dry mucosa	3(1.4)
Itching	3(1.4)
Stroke	3(1.4)
Hypoxic encephalopathy	2(0.9)
Diarrhea	2(0.9)

**Table 3:** Probability of adverse drug reactions based on Naranjo scale

**Type of ADR**	**(%)**	**Naranjo ** **probability ** **scale** ** of ADRs** ** (%)**			
		**Doubtful**	**Possible**	**Probable**	**Highly probable**
Chest pain	62.1%	37.9%			
Hypotension	65.4%			34.6%	
Coughing	87.1%		3.7%	4.7%	
Ecchymose	89.9%		2.3%	7.9%	
Pain in the extremities	89.9%			10.1%	
Nausea and vomiting	89.9%			10.1%	
Bronchospasm	90.1%		1.4%	85%	
Bradycardia	90.3%		9.7%		
Sweating	90.3%			9.7%	
Tachycardia	91.2%		8.8%		
Fever and chill	91.6%				
Ventricular fibrillation	92.6%		7.4%		
Headache	94%		2.3%	3.7%	
Hypertension	94.9%		5.1%		
Bleeding	94.9%				5.1%
Agitation	95.4%		4.6%		
Back pain	95.7%			4.3%	
Abdominal cramp	95.8%		4.2%		
Gastrointestinal bleeding	95.8%				4.2%
Pulmonary edema	96.3%		3.7%		
Epigastric pain	96.3%	3.7%			
Constipation	96.3%		3.7%		
Hematuria	96.7%				3.3%
Hemoptysis	96.7%				3.3%
Allergy and erythema	97.2%			2.8%	
Phlebitis	97.2%			2.8%	
Cardiogenic shock	97.2%	2.8%			
Atrial fibrillation	97.6%		2.3%		
Swelling and edema in the extremities	98.1%			1.9%	
Dry mucosa	98.6%		1.4%		
Itching	98.6%			1.4%	
Stroke	98.6%			1.4%	
Hypoxic encephalopathy	99%		1%		
Diarrhea	99.1%		0.9%		

**Table 4:** Food and drug administration (FDA) classification of severity of adverse drug reactions

**Type of ADR**	**(%)**	**Severity of ADRs**			
		**Mild**	**Moderate**	**Severe**	**Very severe**
Chest pain	62.1%	16.2	21.7		
Hypotension	65.4%	15.7	18.5	0.5	
Coughing	87.1%	4.6	8.3		
Ecchymose	89.9%	3.2	8.3		
Pain in the extremities	89.9%	3.7	6.4		
Nausea and vomiting	89.9%	4.6	5.5		
Bronchospasm	90.1%	1.9	4.2	3.8	
Bradycardia	90.3%	4.6	4.6	0.5	
Sweating	90.3%	9.7			
Tachycardia	91.2%	4.2	4.6		
Fever and chill	91.6%	1.9	6.5		
Ventricular fibrillation	92.6%		2.3	5.1	
Headache	94%	2.3	3.7		
Hypertension	94.9%	2.8	2.3		
Bleeding	94.9%	2.3	0.9	1.9	
Agitation	95.4%		4.6		
Back pain	95.7%	1.5	2.8		
Abdominal cramp	95.8%	1.4	2.8		
Gastrointestinal bleeding	95.8%		2.8	1.4	
Pulmonary edema	96.3%			3.2	0.5
Epigastric pain	96.3%	2.3	1.4		
Constipation	96.3%	0.9	2.8		
Hematuria	96.7%	0.5	0.9	1.9	
Hemoptysis	96.7%		0.5	2.8	
Allergy and erythema	97.2%	0.9	1.9		
Phlebitis	97.2%	0.5	2.3		
Cardiogenic shock	97.2%		0.5	2.3	
Atrial fibrillation	97.6%		0.5	1.9	
Swelling and edema in the extremities	98.1%		1.9		
Dry mucosa	98.6%	1.4			
Itching	98.6%	1.4			
Stroke	98.6%			1.4	
Hypoxic encephalopathy	99%			0.5	0.5
Diarrhea	99.1%	0.4	0.5		

**Table 5:** Correlation between study parameters and adverse drug reactions using Spearman test

**Significant Parameters**	**Correlation Coefficient(r)**	***P*** **-value**
Gender-number of ADRs	0.152	0.025
Gender-incidence of ADR	0.136	0.046
Gender-death	0.212	0.002
Gender-anaphylactic reactions	0.141	0.038
Gender-ecchymosis	0.169	0.012
Gender-headache	0.135	0.047
Number of ADRs-age	0.136	0.045
Number of ADRS -history of poly-pharmacy	0.212	0.002
Number of ADRs –VF	0.205	0.002
Number of ADRs – PE	0.160	0.020
Number of ADRs - hemoglobin deficiency	-0.225	0.001
Number of ADRs -allergy history	0.143	0.036
Number of ADRs - history of anti-coagulant usage	0.163	0.017
Incidence of ADRs - location of MI	0.143	0.047
Incidence of ADR – history of allergy	0.160	0.018
Incidence of ADRs- PE	0.184	0.006
Incidence of ADRs - dyslipidemia	0.174	0.010
Incidence of ADRs - EF	-0.150	0.031
location of MI - EF	-0.142	0.042
Age- EF	-0.210	0.004
location of MI - bradycardia	0.152	0.034
location of MI-tachycardia	0.183	0.012
History of polypharmacy- Incidence of ADRs	0.154	0.023
History of polypharmacy- PE	0.159	0.019
History of polypharmacy-fever	0.147	0.030
History of polypharmacy-ecchymosis	0.145	0.033
ADRs: adverse drug reactions, EF: ejection fraction, MI: myocardial infarction, PE: pulmonary embolism, VF: ventricular tachycardia.		

**Table 6:** The linear regression model between the incidence of adverse drug reactions and independent factors

**Model no.**	**Factor**	**Standardized ** **Beta**	***P*** **-value**	**R** ^2^	**Adjusted R** ^2^	**95% Confidence Interval for B**
**1**	History of allergy	0.234	0.011	0.055	0.046	0.208-1.53
**2**	History of allergy Age	0.248 0.219	0.006 0.014	0.102	0.087	0.272 – 1.57 0.001 - 0.011
**3**	Allergy Age History of PCI	0.251 0.214 0.199	0.005 0.015 0.023	0.142	0.119	0.296 – 1.57 0.001 - 0.011 0.053 – 0.701
**4**	Allergy Age History of PCI Dyslipidemia	0.244 0.237 0.207 0.203	0.005 0.007 0.016 0.019	0.182	0.154	0.282 – 1.53 0.002 - 0.011 0.073- 0.709 0.030 – 0.339

PCI: percutaneous coronary intervention.

**Table 7:** The linear regression model between the number of adverse drug reactions and independent factors.

**Model no.**	**Factor**	**Standardized ** **Beta**	**P-value**	**R** ^2^	**Adjusted R** ^2^	**95% Confidence Interval for B**
**1**	Anticoagulant use	0.319	0.0001	0.102	0.094	0.763-2.58
**2**	Anticoagulant use Hemoglobin	0.280 -0.253	0.001 0.004	0.164	0.150	0.580 – 2.36 -0.318 - -0.062
**3**	Anticoagulant use Hemoglobin Age	0.276 -0.207 0.173	0.002 0.020 0.049	0.192	0.171	0.565 – 2.33 -0.267 - -0.025 0.00 – 0.044

In this research, 54.9% of patients experienced the anterior MI. Inferior and lateral MI were also found in 44.6% and 0.5% of the patients, respectively.

The number of ADRs experienced by patients was between 0 and 8. In average, each patient experienced 2.3 ± 1.7 ADRs. Furthermore, the incidence of ADRs between females and males were 2.6 versus 0.63 indicating the higher rate of ADR (4.2 times more) among female patients.

The most common ADRs were chest pain, hypotension, coughing, and ecchymosis with the frequency of more than fifty percent. The hemorrhagic stroke as the most serious ADR of streptokinase was documented in three patients. In Table 1. the patientsꞌ demographics and clinical data are presented. Also, the frequency of documented ADRs was presented in Table 2. 

According to the Naranjo scale, the most ADRs ranked as “probable” (Table 3). Based on the FDA severity classification of ADRs, the most ADRs were categorized in mild to moderate ADR (Table 4).

The results of Spearman analysis showed that the incidence of ADRs by streptokinase was associated with the female gender (r = 0.136; *p =*0.046), history of allergy (r = 0.160; *p* =0.018), hyperlipidemia (r = 0.174; *p *=0.010), history of pulmonary thromboembolism (PTE) (r = 0.184; *p* =0.006), location of MI (r = 0.143; *p* =0.047), low Ejection fraction (EF) (r = -0.150; *p *=0.031) and history of polypharmacy (r = 0.154; *p *=0.023). Furthermore, the number of ADRs was linked with the age (r = 0.136; *p* =0.045), female gender (r = 0.152; *p* =0.025), incidence of ventricular fibrillation (VF) (r = 0.205;* p *=0.002), history of allergy (r = 0.143; *p* =0.036) and use of anti-coagulant agents (r = 0.163; *p *=0.017) (Table 5).

The linear regression analysis showed the three models of correlation between the incidence of ADRs and study risk factors including age, history of allergy, history of percutaneous coronary intervention and dyslipidemia (Table 6). In the linear regression model, a significant correlation between the number of ADRs and use of anti-coagulant agents, low hemoglobin level and age of patients was documented (Table 7).

The logistic regression analysis showed the history of allergy as the main predictor in the incidence of streptokinase ADRs (Odds ratio: 3.26, 95% CI: 1.48-457.6; *p* =0.026).

## Discussion

This study evaluated 217 patients with acute ST-elevation MI who received streptokinase. The main finding of this research may be that the history of drug allergy was the main predictor in the incidence of ADRs induced by streptokinase. Furthermore, hypotension was one of the most occurred ADR that may be associated with the rapid injection of the drug. Moreover, in this study hemorrhagic stroke and bleeding complications was higher than the other reports. Although, no case of anaphylactic shock was documented.

In our study, the incidence of ADRs by streptokinase was reported 82% that is substantially higher than the other reports. For example, in Devi *et al*. retrospective cohort study and Mohebbi *et al*. prospective study that were conducted in coronary care unit (CCU), the highest ADRs were seen by streptokinase in about 60% (11, 12). However, this rate is similar to our recent study on reteplase with the incidence rate of 85% (13). 

Several studies have described the history of allergy as one of the predisposing factors in occurring ADRs. Hurwits *et al*. (14) in a prospective drug surveillance study of 1160 hospitalized patients showed that there was a significant relation between incidence of ADRs during the hospitalization and history of allergy among 118 patients. In the present study, the logistic regression analysis indicated drug allergy with the odds ratio of 3.26 as a predisposing factor in occurring of ADR by streptokinase. 

One of the most occurred ADR was hypotension that may linked with the rapid injection of the drug. According to the previous studies, hypotension during streptokinase injection is a vasodilatory effect that is occurred by activation of plasmin and producing bradykinin (15, 16). The rate of hypotension during streptokinase therapy was reported between 1-10% that is relatively lower than our study finding (more than 50%) which can reflect the rapid injection of streptokinase (17). In the recent review of Iranian literature, hypotension and arrhythmia were identified as the most frequent ADRs induced by streptokinase that is in agreement with our findings (18). 

Hemorrhagic stroke was the most severe side effect that occurred during hospitalization after the injection of streptokinase by documenting in 3 cases (1.4%). However, the incidence of hemorrhagic stroke in these patients substantially was higher than the other reports. In a prospective and spontaneous reporting-based pharmacovigilance program in Cuba among 792 patients who received streptokinase, the hemorrhagic stroke were reported only in 3 cases (0.3%) (19). In GISI-1 and ISIS-2 trials the incidence of major bleeding such as hemorrhagic stroke was reported between 0.3-0.5% (4, 5). Moreover, in Maggioni *et al*. (20) study, the incidence of various forms of stroke in patients treated with streptokinase was 0.94 %. Many factors including drug related factors such as bleeding complications as well as host related factors such as older age, female gender, anterior MI, history of smoking and hypertension are the main participating factors in occurring stroke (20).

Several studies have described the role of gender in the incidence of ADRs. In the analysis of 48 cohort studies with 513,606 patients in UK, females were more likely to experience ADRs (60% more than males) (21). Also, according to the Hurwits and Seidl *et al.* studies female patients are more likely than men to experience ADRs (13, 22). Similarly, in our study the incidence of ADRs in females was more than males that is in agreement with the earlier reports. 

Moreover, our study indicated that the number of side effects increased along with the increasing age that is consistent with the numerous previous reports. Ageing related decrease in kidney and liver function may be one of the responsible factors (23, 24). Existence of various underlying diseases and poly pharmacy are the other participating factors that put the older patients to experience more ADRs (23, 24). 

The significant correlation between the incidence of ADRs and underlying diseases such as hyperlipidemia, VF, PTE was the other finding of present study that is in line with the other reports, which had indicated the background diseases as a main predictor in the incidence of ADRs (1). 

*Limitations and strengths*

Despite the acceptable sample size of the study (n = 217), the major limitation in such studies is the partially small sample size. In fact, for precise detection and evaluation of all ADRs induced by a special drug a large sample size (more than 10,000) are needed. Second, present study included the relatively weak correlation coefficient that may be linked to this matter. Therefore, the result of this study should be interpreted with a caution. This study also included some key findings as strength points. First, it showed that the patients with history of drug allergy 3.2 times are more vulnerable to show one ADRs by streptokinase. This result could guide clinicians to use streptokinase with a caution in patients with the history of drug allergy. Second, the rate of hemorrhagic stroke in Iranian population is relatively higher than the other reports. This point along with the lower efficacy of streptokinase in the maintaining of thrombolysis in myocardial ischemia (TIMI) III flow may discourage the use of streptokinase by clinicians.

In summary, our study showed that the history of drug allergy is the main predictor in occurring ADRs by streptokinase. Furthermore, streptokinase therapy was associated with a higher rate of hemorrhagic stroke in Iranian population. 
